# Improving Bioactive Characteristics of Small Diameter Polytetrafluoroethylene Stent Grafts by Electrospinning: A Comparative Hemocompatibility Study

**DOI:** 10.3390/bioengineering10040411

**Published:** 2023-03-26

**Authors:** Meltem Avci-Adali, Gerd Grözinger, Vincent Cabane, Michiel Schreve, Hans Peter Wendel

**Affiliations:** 1 Department of Thoracic and Cardiovascular Surgery, University Hospital Tuebingen, Calwerstraße 7/1, 72076 Tuebingen, Germany; 2 Department of Diagnostic and Interventional Radiology, University Hospital Tuebingen, Hoppe-Seyler-Strasse 3, 72076 Tuebingen, Germany; 3 LimFlow SA, 15 Rue Traversière, 75012 Paris, France; 4 Department of Surgery, Northwest Clinics, 1815 JD Alkmaar, The Netherlands

**Keywords:** polytetrafluoroethylene stent graft, electrospinning, hemocompatibility

## Abstract

Polytetrafluoroethylene (PTFE) is a commonly used biomaterial for the manufacturing of vascular grafts and several strategies, such as coatings, have been explored to improve the hemocompatibility of small-diameter prostheses. In this study, the hemocompatibility properties of novel stent grafts covered with electrospun PTFE (LimFlow Gen-1 and LimFlow Gen-2) were compared with uncoated and heparin-coated PTFE grafts (Gore Viabahn^®^) using fresh human blood in a Chandler closed-loop system. After 60 min of incubation, the blood samples were examined hematologically and activation of coagulation, platelets, and the complement system were analyzed. In addition, the adsorbed fibrinogen on the stent grafts was measured and the thrombogenicity was assessed by SEM. Significantly lower adsorption of fibrinogen was measured on the surface of heparin-coated Viabahn than on the surface of the uncoated Viabahn. Furthermore, LimFlow Gen-1 stent grafts showed lower fibrinogen adsorption than the uncoated Viabahn^®^, and the LimFlow Gen-2 stent grafts showed comparable fibrinogen adsorption as the heparin-coated Viabahn^®^. SEM analysis revealed no sign of thrombus formation on any of the stent surfaces. LimFlow Gen-2 stent grafts covered with electrospun PTFE exhibited bioactive characteristics and revealed improved hemocompatibility in terms of reduced adhesion of fibrinogen, activation of platelets, and coagulation (assessed by β-TG and TAT levels) similar to heparin-coated ePTFE prostheses. Thus, this study demonstrated improved hemocompatibility of electrospun PTFE. The next step is to conduct in vivo studies to confirm whether electrospinning-induced changes to the PTFE surface can reduce the risk of thrombus formation and provide clinical benefits.

## 1. Introduction

Biomaterials are widely used in various medical devices, with a strong predominance in the field of cardiovascular indications [1,2]. Among the polymers already accepted as biomaterials, polytetrafluoroethylene (PTFE) is a well-known biocompatible material and is frequently used for the fabrication of cardiovascular implants [2]. Nevertheless, cellular adhesion and growth remain limited, leading to undesirable clinical events, such as neointimal hyperplasia or acute thrombosis. Therefore, several strategies, such as modification of the surface, have been explored to promote in situ endothelialization [3,4,5]. 

In addition to the various chemical modifications that can be made to the surface of polymers, such as passive coating or covalent binding of biofunctional molecules, alternative manufacturing processes, such as electrospinning, have been developed to physically change the surface [6,7]. When applied to PTFE, these techniques can create non-flat structures known as expanded PTFE (ePTFE) or electrospun PTFE. Several studies have already indicated that these modifications of the PTFE structure have a positive impact on cellular and inflammatory responses without modifying the chemistry of the surface [8,9].

These properties are particularly beneficial for the manufacturing of small-diameter prosthetic vascular grafts where clinical complications remain an obstacle to long-term patency [6,10,11]. Expanded PTFE is already widely used for the lining of vascular grafts. However, this biomaterial has some limitations in smaller blood vessels < 5 mm and several efforts have been made to improve the hemocompatibility of the PTFE surface [12], such as coating with hyaluronan [13], tropoelastin [14], and RGD [15,16,17]. Currently, it is often coated with bonded heparin to improve its hemocompatibility and reduce the risk of occlusion [1]. 

Percutaneous deep venous arterialization (pDVA) is a novel procedure for treating patients with end-stage, no-option chronic limb-threatening ischemia (CLTI) where arterial blood is redistributed by an endograft crossing into the veins of the lower extremity [18]. LimFlow^®^ offers a dedicated set of stent grafts for pDVA using a self-expanding nitinol stent with an electrospun PTFE cover for implantation into the arterialized veins and to enable a crossing of the blood from the artery to the veins using a tapered stent graft. Despite good clinical mid-term results, the primary patency of the pDVA may be as low as 40% after 6 months [19]. 

New technologies are needed to improve the biocompatibility and ultimately patency of these long PTFE endografts. Changing the morphology and topography of a material, for example, through electrospinning can influence platelet adhesion and activation [20]. Platelets can sense the extracellular environment via filipodia, which signals the ability for the platelet to spread to the activated form and connect with other platelets. However, fiber curvatures and holes generated by randomly aligned electrospun fibers can prevent the extension of filipodia. Ding [21] and Lamichhane [8] found that electrospun or grooved substrates can reduce platelet adhesion and activation more compared to smooth substrates. Thus, this study aims to evaluate the hemocompatibility of the novel electrospun PTFE in comparison to uncoated and heparin-coated PTFE. 

## 2. Methods

### 2.1. Experimental Setting

All experiments were performed with blood collected from three or eight healthy donors using a modified Chandler-Loop model [22,23]. The closed-loop in vitro model consisted of a thermostated water bath (37 °C) and a rotating unit with attached polyvinyl chloride (PVC) loops. The PVC loops were coated with covalently bonded heparin (CBAS, Carmeda bioactive surface, Medtronic Anaheim, CA, USA) to minimize background activation. The stents under investigation were placed in the loops, which were filled with 12 mL fresh heparinized human whole blood from one donor, then closed into circuits (tubing length 50 cm, ID 4.3 mm). The blood flow within the stents is generated by the rotation of the tubing loop around a horizontal axis. The tubing loops were rotated vertically at 30 rpm in the water bath and the blood was collected in appropriate syringes after 60 min of circulation at 37 °C. The 60 min blood incubation period was chosen to capture the effect of most reactions without losing any initial information due to unnecessarily prolonged exposure.

### 2.2. Stent Types

**Figure 1:**
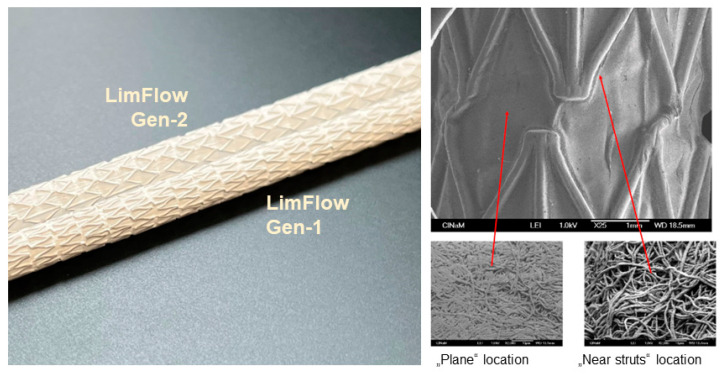
Images of LimFlow stent grafts. Photographic images showing LimFlow Gen-1 and Gen-2 stent grafts (**left**), and SEM images showing the surface of the LimFlow Gen-2 stent grafts (**right**).

Using the above setting, three series of experiments were performed comparing LimFlow Gen-1 and Gen-2 stent-grafts (LimFlow, Paris, France) covered with electrospun PTFE to Gore^®^ Viabahn^®^ coated and uncoated endoprostheses (W. L. Gore & Associates, Flagstaff, AZ, USA). Electrospun PTFE was prepared in the form of sheets, which were then wrapped externally and internally around and inside the bare metal stent. The difference between the LimFlow Gen-1 and Gen-2 stent grafts is the shape of the nitinol struts and the number of electrospun PTFE layers (five layers for the Gen-1, and three layers for Gen-2 stent grafts) (Figure 1). However, the nature of the material in contact with the blood was identical. Similarly, the only difference between the Gore^®^ Viabahn^®^ coated and uncoated endoprostheses consists in the presence of a Propaten^®^ bioactive surface (heparin coating). 

The first series of experiments was a pilot study (A) comparing three samples of uncoated Viabahn^®^ endoprostheses (6.0 × 50 mm), heparin-coated Viabahn^®^ endoprostheses (5.0 × 50 mm), and LimFlow Gen-1 stent grafts (5.5 × 60 mm). The primary objective of this pilot study was to assess the feasibility of the modified Chandler-Loop model for hemocompatibility testing. Afterwards, a confirmatory study (B) was performed to compare eight samples of uncoated Viabahn^®^ endoprostheses (5.0 × 50 mm), heparin-coated Viabahn^®^ endoprostheses (5.0 × 50 mm), and LimFlow Gen-1 stent grafts (5.5 × 50 mm). The main objective of this study was to compare the LimFlow Gen-1 stent graft with the Viabahn^®^ endoprostheses. A final study (C) was performed to compare eight samples of heparin-coated Viabahn^®^ endoprostheses (5.0 × 50 mm), LimFlow Gen-1 stent grafts (5.5 × 60 mm), and LimFlow Gen-2 stent grafts (5.5 × 60 mm). The main objective of this study was to compare the LimFlow Gen-1 and Gen-2 stent grafts.

The blood from each donor was tested in parallel with each of the stents. The type and number of stents compared in each experiment are summarized in Table 1. All the test items were final products sterilized with EtO. The 150 mm long Viabahn^®^ endoprostheses and LimFlow stent grafts were cut into three 50 mm shorter pieces to obtain test items of comparable size, with all diameters between 5 and 6 mm, and all lengths between 50 mm and 60 mm.

### 2.3. Blood Preparation

Blood was obtained by venipuncture from different donors aged between 20 years and 50 years with a 1:1 gender ratio male to female. Smokers or volunteers taking drugs in the last two weeks were excluded. The blood was anticoagulated with 1 IU/mL sodium heparin (Ratiopharm GmbH, Ulm, Germany) to avoid excessive coagulation activation. In addition to the test materials (stent grafts), blood samples were taken for baseline control (reference blood without contact with the test material and incubation), and negative control (blood without test material after incubation). Immediately after blood sampling (baseline control) and 60 min after incubation, blood was transferred into monovettes containing EDTA for detection of blood cell counts using a Micros 60 cell counter (ABX Hematology, Montpellier, France) and complement activation marker sC5b-9. Monovettes containing citrate were used to analyze thrombin-antithrombin complex (TAT). To analyze β-thromboglobulin (β-TG) concentrations, blood was transferred to citrate-theophylline-adenosine-dipyridamole (CTAD) containing monovettes (BD Biosciences Inc.). EDTA and CTAD monovettes were centrifuged at 2500× *g* for 20 min at 4 °C. Citrate blood monovettes were centrifuged at 1800× *g* for 18 min at RT. The plasma of each sample was shock frozen in liquid nitrogen and stored at −20 °C (EDTA and citrate plasma) or −80 °C (CTAD plasma) until further investigations.

### 2.4. Detection of Activation Markers

The analyses of the above samples were performed according to ISO 10993-4 by testing the thrombogenicity, coagulation, platelet numbers and activation, and complement activation. In addition, hematology analyses were performed to further assess the hemocompatibility of the test materials. 

#### 2.4.1. Coagulation

Contact of blood with materials initiates the intrinsic coagulation pathway via factors XII, XI, IX, and finally, Factor X, which modulates the conversion of prothrombin (Factor II). During the reaction of thrombin formation, prothrombin is cleaved into fragment 1.2 and active thrombin. The thrombin formed is inactivated by complex formation with antithrombin to form the TAT. Thus, the TAT concentration in plasma is an excellent marker for coagulation activation and was measured immunochemically using ELISA (Siemens Healthcare Diagnostics Products, Marburg, Germany).

#### 2.4.2. Platelets

Contact of blood with material surfaces can lead to platelet activation and alteration, resulting in loss of platelet functionality, which was quantified by platelet counts. Platelet activation was further assessed by measurements of β-TG, a protein that is stored in platelets α-granules and released after platelet activation, using ELISA (Diagnostica Stago/Roche, Asnières, France).

#### 2.4.3. Hematology

White and red blood cells were counted using a Micros 60 cell counter (ABX Hematology, Montpellier, France), and the concentration of hematocrit and hemoglobin was measured. Hemolysis, an indicator for red blood cell destruction, was quantified using a colorimetric assay by measuring free plasma hemoglobin using a cyan hemoglobin test.

#### 2.4.4. Complement System

Complement activation in the samples was assessed by quantification of the terminal complement complex sC5b-9 using ELISA (Quidel, San Diego, CA, USA). 

### 2.5. Analysis of Thrombogenicity

SEM (Zeiss, EVOLS 10, Oberkochen, Germany) was used to analyze the cellular adhesion and fibrin generation on the stent surfaces. In addition to this qualitative analysis, surface-bound plasma proteins were characterized using a modified ELISA technique with fibrinogen-binding antibodies and fibrinogen adsorption was quantified by optical density.

### 2.6. Statistics

Statistical justification of the sample size was completed according to ISO 10993-4:2017, leading to a sample size of eight samples per study following the initial pilot study. Results were presented as bar diagrams representing means and standard deviations (SD). One-way ANOVA followed by Bonferroni’s multiple comparison test was performed to evaluate the statistical significance of the differences of normally distributed data and Friedman test followed by Dunn’s multiple comparison test was performed to determine differences of not normally distributed data. Values of *p* < 0.05 were considered statistically significant.

## 3. Results

### 3.1. Analysis of Fibrinogen Adsorption

**Figure 2:**
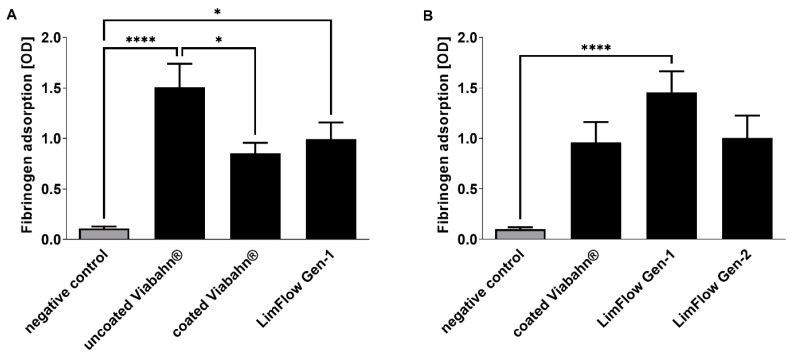
Analysis of fibrinogen adsorption to the graft surfaces. Detection of adsorbed fibrinogen in (**A**) experiment series B and (**B**) experiment series C. The data are shown as mean + SD. Statistical analysis was performed using the Friedman test followed by Dunn’s multiple comparison test. *n* = 8, (* *p* < 0.05, **** *p* < 0.0001).

After incubation of the stents with blood, adsorption of fibrinogen on the surface was quantified in experimental series B and C (Figure 2). A significantly increased fibrinogen adsorption was observed on uncoated Viabahn^®^ and LimFlow Gen-1 stent grafts compared to the negative control (Figure 2A). However, LimFlow Gen 1 stent grafts showed lower fibrinogen adsorption than the uncoated Viabahn^®^. Heparin-coated Viabahn^®^ significantly reduced fibrinogen adsorption compared with uncoated Viabahn^®^. In experimental series C, the LimFlow Gen-2 stent graft showed comparable fibrinogen adsorption as the heparin-coated Viabahn^®^ (Figure 2B).

### 3.2. Analysis of Coagulation Activation

**Figure 3:**
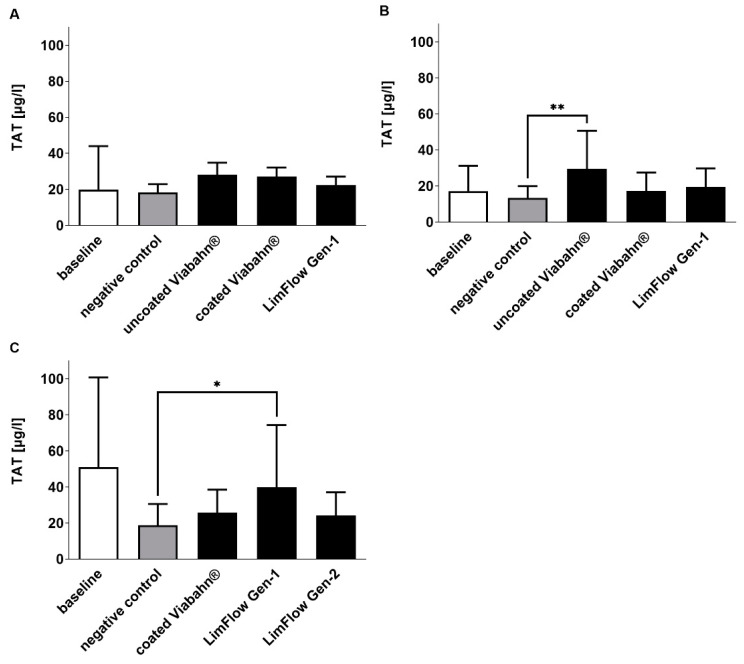
Analysis of TAT concentrations in plasma after the incubation of stent grafts with fresh human blood. (**A**) Pilot study (*n* = 3), (**B**) Experiment series B (*n* = 8), (**C**) Experiment series C (*n* = 7). The data are shown as mean + SD. Statistical analysis was performed using the Friedman test followed by Dunn´s multiple comparison test. *N* = 8, (* *p* < 0.05, ** *p* < 0.01).

The activation of coagulation was analyzed by the detection of TAT concentrations (Figure 3). Very low levels were measured in all stent groups (ranging from 17.3 ± 10.2 µg/L to 40.0 ± 34.4 µg/L). However, compared to the negative control, uncoated Viabahn^®^ (Figure 3B) and LimFlow Gen-1 stent grafts (Figure 3C) showed significantly higher TAT levels. LimFlow Gen-2 showed comparable to heparin-coated Viabahn^®^ no significant differences to negative control and demonstrated an improvement in reduction in coagulation activation. 

### 3.3. Analysis of Platelet Numbers and Activation

**Figure 4:**
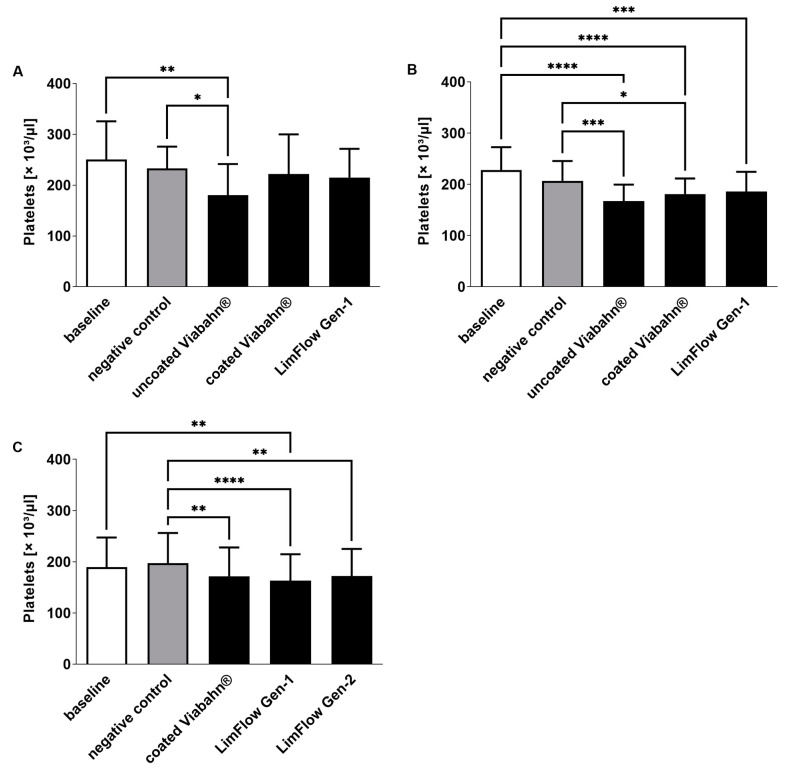
Analysis of platelet counts after the incubation of fresh human blood with test items for 60 min at 37 °C. (**A**) Pilot study (*n* = 3), (**B**) Experiment series B (*n* = 8), (**C**) Experiment series C (*n* = 8). Whole human blood without incubation and contact to test material served as baseline, and blood without test material but incubation as negative control. The data are shown as mean + SD. Statistical differences were determined using one-way ANOVA for repeated measurements followed by Bonferroni’s multiple comparison test. (* *p* < 0.05, ** *p* < 0.01, *** *p* < 0.001, **** *p* < 0.0001).

**Figure 5:**
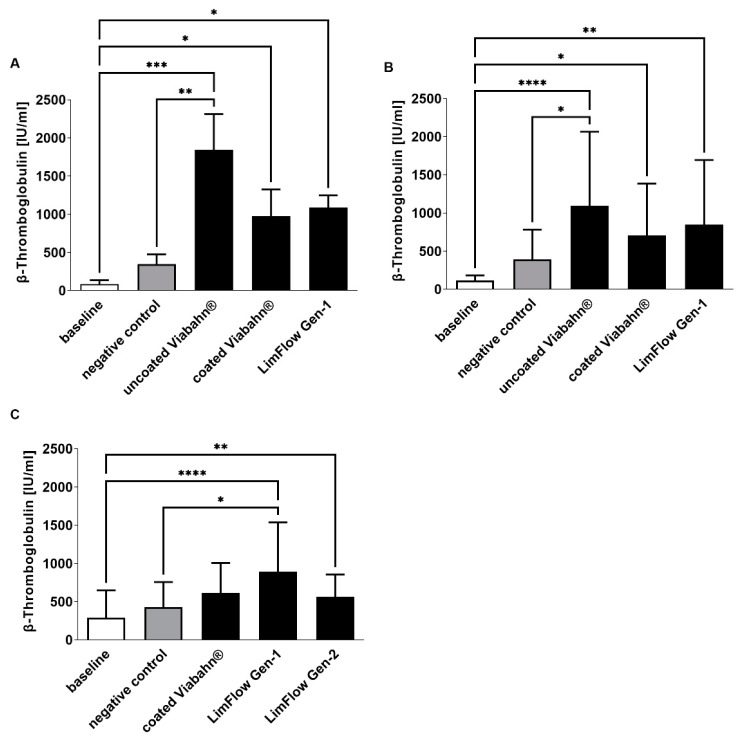
Analysis of platelet activation by measuring β-TG concentration after the incubation of fresh human blood with test items for 60 min at 37 °C. (**A**) Pilot study (*n* = 3), Statistical differences were determined using one-way ANOVA for repeated measurements followed by Bonferroni’s multiple comparison test. (**B**) Study B (*n* = 8), (**C**) Study C (*n* = 8). Statistical analysis was performed using the Friedman test followed by Dunn´s multiple comparison test. Whole human blood without incubation and contact to test material served as baseline and blood without test material, but with incubation as the negative control. The data are shown as mean + SD. (* *p* < 0.05, ** *p* < 0.01, *** *p* < 0.001, **** *p* < 0.0001).

The platelet counts slightly decreased in all stent groups compared to negative controls (Figure 4), reflecting adherence of platelets to the stent surface. The highest platelet activation, as evidenced by an increase in β-TG, was observed with the uncoated Viabahn^®^ endoprosthesis (Figure 5A,B). While the LimFlow Gen 1 stent graft led to significantly higher β-TG values compared with the negative control in experimental study C (Figure 5C), no statistical differences were detected in the pilot study and experimental study B. The LimFlow Gen 2 stent graft showed comparable values to the coated Viabahn^®^ endoprosthesis and the β-TG values were not significantly different from those of the negative control.

### 3.4. Hematological Analyses

**Figure 6:**
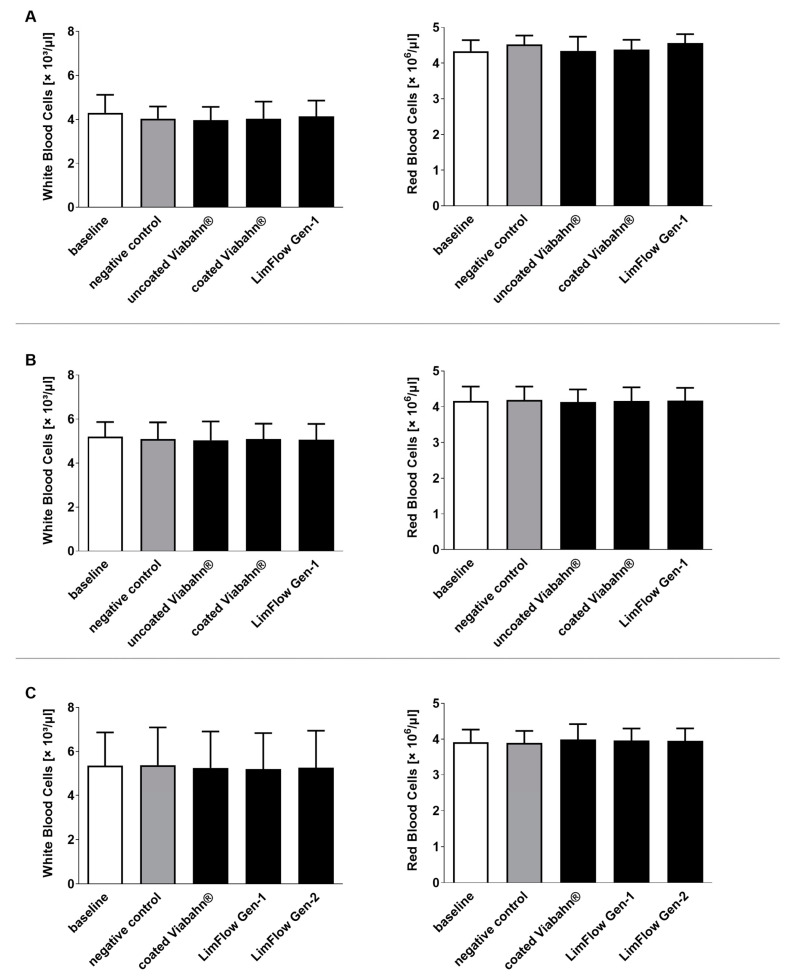
Analysis of white and red blood cell counts after the incubation of fresh human blood with test items for 60 min at 37 °C. (**A**) Pilot study (*n* = 3), (**B**) Study B (*n* = 8), (**C**) Study C (*n* = 8). Whole human blood without incubation and contact to test material served as baseline and blood without test material, but with incubation as the negative control. The data are shown as mean + SD.

**Figure 7:**
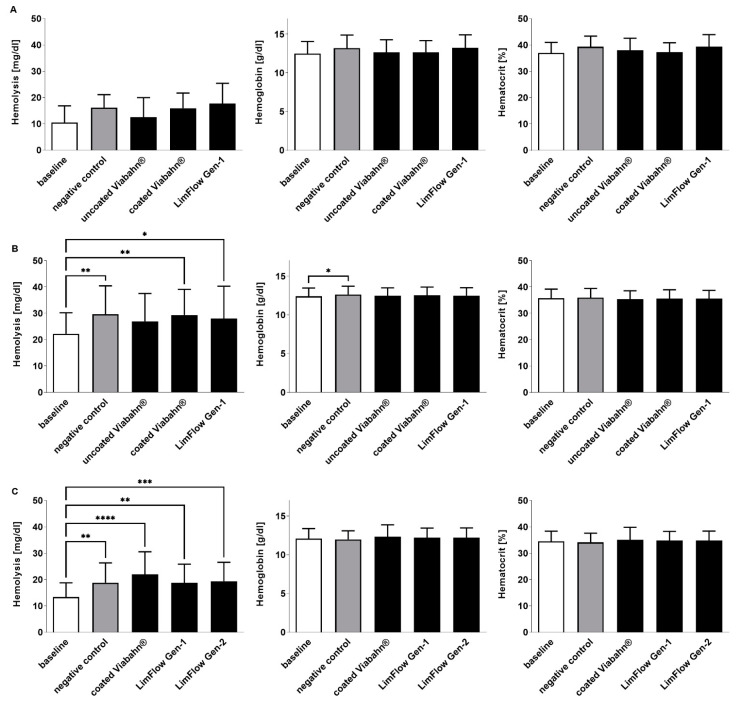
Analysis of hemolysis, hemoglobin, and hematocrit levels after the incubation of fresh human blood with test items for 60 min at 37 °C. (**A**) Pilot study (*n* = 3), (**B**) Study B (*n* = 8), (**C**) Study C (*n* = 8). Whole human blood without incubation and contact to test material served as baseline, and blood without test material, but with incubation as the negative control. The data are shown as mean + SD. Statistical differences were determined using one-way ANOVA for repeated measurements followed by Bonferroni’s multiple comparison test. (* *p* < 0.05, ** *p* < 0.01, *** *p* < 0.001, **** *p* < 0.0001).

During the circulation period, white and red blood cell counts remained stable in all three studies (Figure 6). Significant changes compared to the negative control were also not observed in hemolysis, hematocrit, and hemoglobin levels after the incubation of stents with blood (Figure 7). The slightly increased hemolysis (Figure 7A) after the circulation is possibly caused due to the rotation of the blood in the Chandler loop.

### 3.5. Complement System

**Figure 8:**
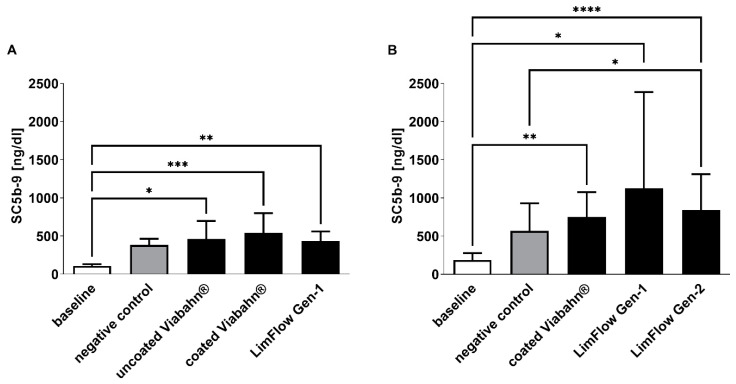
Analysis of complement activation by detection of SC5b-9 concentration. Detection of SC5b-9 in (**A**) experiment series B and (**B**) experiment series C. The data are shown as mean + SD. Statistical analysis was performed using the Friedman test followed by Dunn´s multiple comparison test. *n* = 8, (* *p* < 0.05, ** *p* < 0.01, *** *p* < 0.001, **** *p* < 0.0001).

As expected, baseline blood samples showed the lowest concentrations in complement sC5b-9 (108 ± 22 ng/mL and 185 ± 93 ng/mL on average) (Figure 8). The blood circulation in the modified Chandler loop with all test stents significantly increased the complement activation. However, in comparison to the negative control, slightly higher complement activation was detected only after the incubation of blood with LimFlow Gen 2 stent graft (Figure 8B).

### 3.6. Thrombogenicity

**Figure 9:**
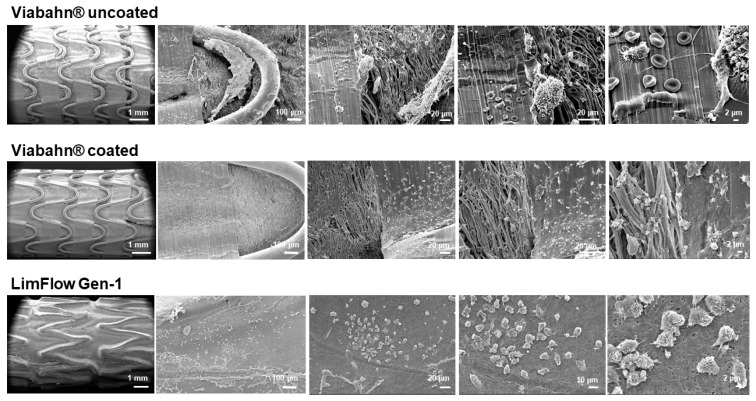
Analysis of the blood-contacting surface of stent grafts using SEM. Detection of the thrombogenicity in experiment series A. Representative images from one donor are shown after the blood contact (*n* = 3).

**Figure 10:**
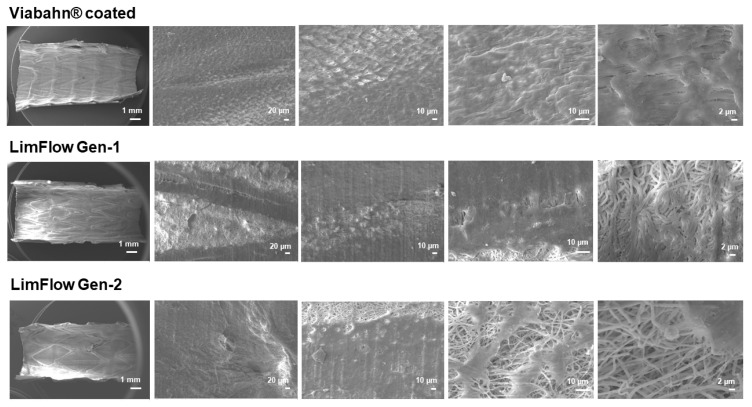
Analysis of the blood-contacting surface of stent grafts using SEM. Detection of the thrombogenicity in experiment series C. Representative images from one donor are shown after the blood contact (*n* = 8).

After circulation in the Chandler loop, cell deposition and protein adsorption were found on the surface of all tested stents. The SEM analyses revealed a few areas of fibrin fibers on all stent surfaces, and slight sticking of platelets and other blood cells (Figure 9 and Figure 10). However, no signs of real thrombus formation could be detected on any stent surface.

## 4. Discussion

In this study, we evaluated the hemocompatibility of novel small-diameter (5 to 6 mm) electrospun PTFE stent grafts in comparison with uncoated ePTFE and heparin-coated ePTFE vascular grafts (Gore^®^ Viabahn^®^). The experiments were performed using a modified Chandler loop model, which allowed an extensive and comparative characterization of the hemocompatibility properties of the different LimFlow stent grafts and Gore^®^ Viabahn^®^ endoprostheses. 

During the initial pilot study with three samples, parameters, including thrombogenicity, coagulation, platelets, and hematology, could be properly assessed. These preliminary results confirmed the validity of the modified Chandler loop model for hemocompatibility testing and led to two subsequent series of experiments. In the second series of experiments (comparing uncoated Viabahn^®^, heparin-coated Viabahn^®^, and LimFlow Gen-1 stent grafts), all statistically significant differences were in favor of the heparin-coated Viabahn^®^ and LimFlow Gen-1 over the uncoated Viabahn^®^. In the third series of experiments (comparing heparin-coated Viabahn^®^, LimFlow Gen-1, and LimFlow Gen-2 stent grafts), the statistically significant differences were in favor of heparin-coated Viabahn^®^ and LimFlow Gen-2 versus LimFlow Gen-1 stents.

Heparin coating is a well-known technique for modifying the surface of cardiovascular implants and improving the hemocompatibility of devices [24,25]. Our study confirmed that this technique was efficient when applied to the Viabahn^®^ endoprosthesis and that the heparin-coated Viabahn^®^ performed better than the uncoated Viabahn^®^. Specifically, less adhesion of fibrinogen to coated Viabahn^®^ was observed compared to uncoated Viabahn^®^. In addition, the activation of coagulation and platelets was reduced. Overall, these experiments (first (A) and second series (B)) showed that the heparin-coated Viabahn^®^ exhibited better hemocompatibility than the uncoated Viabahn^®^, as expected. Similarly, the third series of experiments (C) also indicated that the LimFlow Gen-2 performed better than the LimFlow Gen-1 in terms of reduction in coagulation (TAT) and platelet (β-TG) activation and fibrinogen adsorption. Although higher complement activation was observed compared to the negative control, the hemocompatibility of the LimFlow Gen-2 stent grafts was comparable to heparin-coated Viabahn^®^ prosthesis and there were no significant differences. It should also be noted that LimFlow Gen-1 stent grafts covered with electrospun PTFE performed better than uncoated Viabahn^®^. 

However, the main objective of this work was to evaluate the electrospun PTFE as another approach to influence cellular responses and to assess the impact of this innovative material on the hemocompatibility characteristics of the implant. The results presented above indicate that this modification of the PTFE structure led to a similar hemocompatibility as the heparin-coated Viabahn^®^, confirming the advantages of this technique and the bioactive properties of electrospun PTFE.

This innovative approach, which does not require heparin coating, offers potentially clinically relevant advantages over the use of heparin-coated vascular implants. First, the increased hemocompatibility of electrospun PTFE is an intrinsic property of the biomaterial. In contrast, heparin coating is inherently nondurable since the body contains heparanase, which can degrade heparin. Thus, in future studies, it would be interesting to investigate whether electrospun PTFE provides durable hemocompatibility properties in comparison. Second, it is known that patients pre-exposed to heparin may develop an adverse reaction to heparin, such as heparin-induced thrombocytopenia (HIT) [26,27]. Therefore, providing a biomaterial that has similar hemocompatibility characteristics without exposing patients to another molecule with potentially immunogenic properties has clear clinical benefits.

The heparin-coated Viabahn endoprosthesis is considered the “gold standard” of endoprosthesis for peripheral arterial use. There is a well-established body of evidence regarding the clinical efficacy and good mid- to long-term patency rates [28,29].

**Figure 11:**
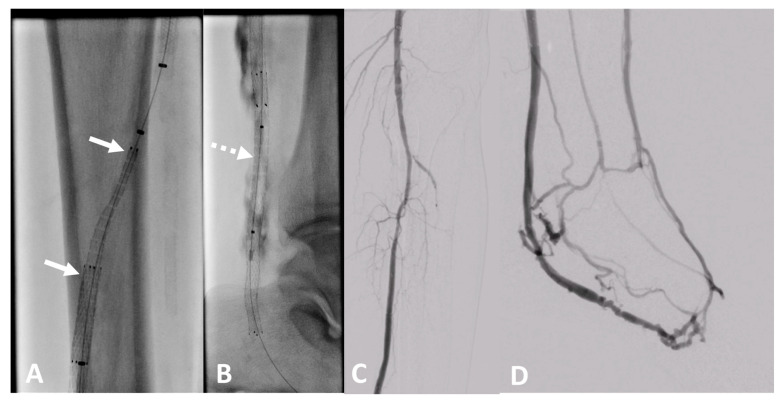
Clinical example of a patient treated at University Hospital Tuebingen with percutaneous deep venous arterialization (pDVA). The aim of this intervention is the redistribution of arterial blood to the foot via the tibial veins as a conduit. (**A**) Implantation of a dedicated tapered LimFlow endograft for crossing from the posterior tibial artery to a tibial vein (white arrows). (**B**) Straight LimFlow endografts in the tibial vein covering side branches until the foot (dotted arrow). (**C**) Angiographic of the proximal lower leg image after implantation of the LimFlow endografts. (**D**) Final angiographic result of the arterialized venous foot arch after successful pDVA.

In recent years, pDVA emerged as a novel treatment option for CLTI patients without an option for conventional angioplasty or bypass caused by an absence of pedal vessels as a target for recanalization. For this procedure, long stent grafts are needed to redirect the arterial blood to the venous vessels of the foot to establish pressurization of the existing small venous vessels of the foot with oxygen-rich blood. The LimFlow stent grafts are dedicated to this indication [30] (Figure 11). However, the long-term experience with regard to patency is currently limited [31].

The results of this in vitro study suggest that the novel LimFlow stent grafts show improved properties compared to the uncoated Viabahn stent, similar to the established Viabahn stent graft with heparin coating. This might enable better long-term patency of Limflow stent grafts used for pDVA in the future. However, this hypothesis has to be proven in further clinical studies.

In terms of study limitations, it should be noted that the experiments were performed using an ex vivo model, which does not fully reflect the in vivo situation. As mentioned above, there were some minor differences in the lengths and diameters of the stent grafts tested, resulting in a different surface area of the material that came into contact with the blood. In addition, blood was obtained from different donors, resulting in interindividual variations between measurements and large standard derivations that may affect the statistical significance of the data. 

## 5. Conclusions

This study compared the hemocompatibility of uncoated or heparin-coated ePTFE prostheses and two variants of electrospun PTFE stents (LimFlow Gen 1 and Gen 2 stent grafts) were compared. As expected, heparin-coated ePTFE prostheses resulted in improved hemocompatibility in terms of reduced adhesion of fibrinogen, and platelet and coagulation activation (assessed by β-TG and TAT concentrations) compared with non-coated ePTFE prostheses. Electrospun PTFE stent grafts, particularly LimFlow Gen 2 stent grafts, exhibited bioactive characteristics at molecular level and revealed high hemocompatibility in terms of reduced adhesion of fibrinogen, and platelet and coagulation activation (assessed by β-TG and TAT concentrations) similar to heparin-coated ePTFE prostheses. 

Changes in surface and bioactive properties have been reported in the literature when electrospinning technique was used to manufacture PTFE-based materials. Our study confirmed the biological properties of electrospun PTFE and demonstrated improved hemocompatibility. As a next step, in vivo studies should be performed to confirm these findings and determine whether these bioactive properties provide clinical benefit when used in a stent graft.

## Figures and Tables

**Table 1:** Type and number of stents.

Series of Experiments	LimFlow Gen-1	LimFlow Gen-2	Viabahn^®^ Uncoated	Viabahn^®^ Coated	Baseline (Reference)	Negative Control
Series A (pilot)	3		3	3	3	3
Series B	8		8	8	8	8
Series C	8	8		8	8	8

## Data Availability

The analyzed data sets generated during the study are available from the corresponding author upon reasonable request.
